# c-Met overexpression in inflammatory breast carcinomas: automated quantification on tissue microarrays

**DOI:** 10.1038/sj.bjc.6603569

**Published:** 2007-01-23

**Authors:** S Garcia, J-P Dalès, J Jacquemier, E Charafe-Jauffret, D Birnbaum, L Andrac-Meyer, M-N Lavaut, C Allasia, S Carpentier-Meunier, P Bonnier, C Charpin-Taranger

**Affiliations:** 1 Department of Pathology, Centre Hospitalo-Universitaire Nord, Marseille, France; 2 Department of Biopathology, Institut Paoli Calmettes, Marseille, France; 3 UMR 599 INSERM, Institut Paoli Calmettes, Marseille, France; 4 Faculté de Médecine Timone, Marseille, France; 5 Department of Gynecology and Breast Oncology, Centre Hospitalier Universitaire de la Conception, Centre Hospitalier Privé Beauregard, Marseille, France

**Keywords:** c-Met, immunocytochemistry, tissue microarray, image analysis

## Abstract

Inflammatory breast carcinoma (IBC) is a rare but aggressive tumour associated with poor outcome owing to early metastases. Increased expression of c-Met protein correlates with reduced survival and high metastatic risk in human cancers including breast carcinomas and is targetable by specific drugs, that could potentially improve the prognosis. In the present study, we compared c-Met expression in IBC (*n*=41) and non-IBC (*n*=480) immunohistochemically (Ventana Benchmark autostainer) in two tissue microarrays (TMA) along with PI3K and E-cadherin. The results were quantified through an automated image analysis device (SAMBA Technologies). We observed that (i) c-Met was significantly overexpressed in IBC as compared with non-IBC (*P*<0.001), (ii) PI3K was overexpressed (*P*<0.001) in IBC, suggesting that the overexpressed c-Met is functionally active at least through the PI3K signal transduction pathway; and (iii) E-cadherin was paradoxically also overexpressed in IBC. We concluded that overexpressed c-Met in IBC constitutes a potential target for specific therapy for the management of patients with poor-outcome tumours such as IBC. Automated image analysis of TMA proved to be a valuable tool for high-throughput immunohistochemical quantification of the expression of intratumorous protein markers.

Inflammatory breast cancers (IBC) are very aggressive tumours that most often develop in young women and are associated with poor 5-year survival ranging from 30 to 50% (Jaiyesimi *et al*, 1992).

Response to primary chemotherapy including anthracycline-based neo-adjuvant therapy is a strong but limited indicator of survival (Buzdar *et al*, 1995). Clinically, IBC are characterised by rapid growth, extensive axillary lymph node metastases and remote metastases at the time of initial diagnosis. Microscopically, the presence of tumour emboli within dermal lymphatic channels (associated with skin erythema and/or oedema) constitutes a major pathologic hallmark of the disease (Tavassoli *et al*, 2003; Bertucci *et al*, 2004; Charafe-Jauffret *et al*, 2004; Gasparini *et al*, 2005). In IBC, genes shown as discriminant for aggressiveness are associated with various cellular functions, in particular cell motility, angiogenesis and adhesion (Bertucci *et al*, 2004; Charafe-Jauffret *et al*, 2004). The role potential of angiogenesis on tumour growth and dissemination prompted a search for therapeutic agents targeting endothelial cells to block angiogenesis and *in vivo* tumour growth. Several agents have been assessed in experimental studies (Pennacchietti *et al*, 2003; Charafe-Jauffret *et al*, 2004), but preliminary results of the clinical studies suggest that anti-angiogenic therapy through a single agent is not highly effective in advanced tumours (Pennacchietti *et al*, 2003; Gasparini *et al*, 2005). Recent studies using a combination of antivascular endothelial growth factor and other therapeutic agents have renewed interest in this therapeutic strategy (Birchmeier *et al*, 2003; Pennacchietti *et al*, 2003; Bertucci *et al*, 2004; Charafe-Jauffret *et al*, 2004; Christensen *et al*, 2005; Gasparini *et al*, 2005).

Some of these studies have shown that combination of selective antiangiogenic and c-Met inhibitors could improve therapeutic efficiency, resulting in a reduction of both tumour growth and metastasis. This observation is based on the fact that hypoxia promotes spread of cancer cells toward a more oxygenated environment in distant tissues through a transcriptional activation of *c-met* proto-oncogene (Pennacchietti *et al*, 2003; Gasparini *et al*, 2005).

The *c-met* proto-oncogene encodes for the high-affinity thyrosin kinase receptor for hepatocyte growth factor (HGF) or scatter factor (SF). c-Met and HGF play a major role in cell migration, morphogenic differentiation, organisation of three-dimensional tubular structures, cell growth and angiogenesis (Birchmeier *et al*, 2003; Pennacchietti *et al*, 2003; Christensen *et al*, 2005). Deregulation of c-Met and HGF have been shown to correlate with a poor outcome in several major human tumours, particularly breast carcinomas (Beviglia *et al*, 1997; Ghoussoub *et al*, 1998; Camp *et al*, 1999; Nakipoulou *et al*, 2000; Tolgay Ocal *et al*, 2003; Christensen *et al*, 2005; Lee *et al*, 2005). In human carcinomas, the receptor tyrosin kinase c-Met becomes activated through several mechanisms, including (i) overexpression and constitutive kinase activation in the presence or absence gene amplification, (ii) paracrine and autocrine activation, (iii) upregulation by mutation or epigenetic mechanisms such as secreted growth factors, tumour hypoxia and activation of other oncogenes (Birchmeier *et al*, 2003; Pennacchietti *et al*, 2003; Christensen *et al*, 2005). Various c-Met inhibitors have recently been developed and experimentally evaluated as potential drugs for treatment of human cancers (Maulik *et al*, 2002; Trusolino and Comoglio, 2002; Ma *et al*, 2003; Christensen *et al*, 2005). Thus, overexpression of c-Met can be targeted by specific inhibitors (Maulik *et al*, 2002; Trusolino and Comoglio, 2002; Ma *et al*, 2003; Christensen *et al*, 2005), and immunohistochemically detected overexpression within tumour biopsies at the time of diagnosis may be relevant for patient management. c-Met expression has never been specifically evaluated in IBC. As tumour cell dissemination to distant sites and metastases are responsible for early patient death, we hypothesised that c-Met could be overexpressed and significantly involved in the process of IBC metastasis and that specific therapy could improve the prognosis for these patients.

In this study, our goal was to evaluate c-Met expression in IBC, parallel with phosphatidylinositol-3-kinase PI3K transductal signal expression. E-cadherin expression was also evaluated, in view of previous results unexpectedly showing overexpression in IBC and subsequent paradoxically increased cell–cell adhesion in highly metastatic tumours (Charafe-Jauffret *et al*, 2004). We investigated c-Met immunocytochemical expression in 41 IBC and compared it with that in a control of 480 not otherwise specified ductal breast carcinomas. For this purpose, we constructed tissue microarrays (TMA) (Kallioniemi *et al*, 2001) specifically dedicated to IBC and 480 non-IBC of various grades, sizes and nodal status, and quantified c-Met immunoprecipitates within the tumour TMA using specific software recently developed for image analysis (SAMBA 2050).

## MATERIALS AND METHODS

### Breast tumour samples and patient characteristics

Inflammatory breast carcinoma samples were obtained from 41 patients who had undergone initial surgery in the Hôpital de la Conception (PB). Tissue fragments were processed in the Pathology Department, Hôpital Nord (SG, CC, LA, SCM). Tumours were selected according to the pTNM classification as pT4d with superficial dermal lymphatic invasion within a skin biopsy sampled at the time of initial diagnosis assessed on surgical tumour biopsy. All tumours selected as IBC were axillary node-positive, oestrogen- and progesterone-receptor-negative, as evaluated by immunohistochemistry (with positivity cut-off value of 1%), P53-positive and HER-2-positive FISH amplification/Ventana Benchmark ⩾6) grade 3 ductal carcinomas with inflammatory stroma and prominent vascular invasion. The IBC were compared with 480 non-IBC ductal breast carcinomas selected from our archival tumour paraffin blocks. Tumour size varied from 7 to 65 mm (m=23 mm, s.d.±8). All tumours were ductal carcinomas not otherwise specified; other subtypes, particularly lobular and medullary carcinomas present in our files, were excluded because of their specific reactivity with regard to E-cadherin. Ductal carcinomas were grade 1 (103 out of 480), grade 2 (270 out of 480) and grade 3 (107 out of 480), and 312 of the patients were node-negative. All tumour biopsies used for TMA construction were fixed in buffered formalin, paraffin-embedded and stored at controlled temperature (18–22°C).

### Tissue microarrays construction

Tissue microarrays were prepared as previously described (Ginestier *et al*, 2002; Charpin *et al*, 2004). Briefly, for each tumour, two representative tumour areas were delineated by circling within tissue sections (SG, CC, JD, SCM) appropriate areas with a permanent black pen on H&E-stained paraffin sections, to guide the technicians' punches of cores within the primary paraffin block. Cores were sampled using the ALPHELYS Arraying Device (ALPHELYS, 78370 Plaisir, France). Core cylinders of 0.6 mm diameter, punched from the donor block, were then deposited in the recipient paraffin block. Tissue microarray sections (4 *μ*m thick) were cut 24 h before immunohistochemical processing.

### Immunohistochemical automated procedure

The IHC procedure was performed with a Ventana Benchmark autostainer using the procedure previously reported (Charpin *et al*, 2004; Dalès *et al*, 2004, 2005) and specific antibodies: mouse monoclonal anti-c-Met (clone 8F11, diluted 1:20) was obtained from Novocastra (TEBU, France); polyclonal rabbit anti-PI3K p110 *α* (diluted 1:10) from Cell Signaling (Ozyme, France); monoclonal mouse anti E-cadherin (clone 4A 2C7, diluted 1 : 100) from Clinisciences (France).

Slides were incubated for 32 min at 37°C with specific primary antibodies.

Diaminobenzidine or 3-amino-9-ethylcarbazole were used as chromogens and slides were counterstained with haematoxylin before mounting. Both chromogens used on regular full sections before TMA testing gave concordant results in terms of both surfaces and intensities of immunostaining. Negative controls were obtained by omitting the specific primary antibodies.

### Preliminary validation of immunodetection and TMA

#### Comparison of c-Met immunoexpression in paraffin and frozen sections

c-Met immunoexpression in 120 paraffin sections was compared with that in 120 frozen sections from tissue blocks sampled in the same tumours. In a preliminary study, we observed that c-Met expression in frozen samples of breast carcinoma correlated with poor survival (unpublished data). Comparison of c-Met immunostaining by semiquantitative evaluation and in paraffin sections by Ventana Benchmark device antigen retrieval gave similar results and immunostaining patterns were significantly correlated in both substrates (Spearman's correlation coefficient *ρ*=0.694, *P*<0.01).

#### Comparison of c-Met expression in different cores punched from the same tumour

Evaluation of c-Met immunoexpression in 50 cases of breast carcinoma selected for TMA showed similar immunostaining in multiple core samples from different areas (but of similar cellularity) of the donor blocks (Spearman's correlation coefficient *ρ*=0.784, *P*<0.01). This indicates that a limited number (two in the present study) of cores was sufficient when areas in the donor blocks were appropriately delineated and representative of tumours.

#### Comparison of semiquantitative analysis and quantitative image analysis

A semiquantitative approach was used to classify immunoexpression as negative or positive. The latter were further subclassified into weak (1+), intermediate (2+) or intense (3+) staining, based on the intensity of immunoreaction. This approach gave results that were significantly correlated (Spearman's correlation coefficient *ρ*=0.698, *P*<0.01) with the densitometric quantification of immunoreactivity assessed by SAMBA image analysis.

### Image analysis

Tissue microarray analysis using the SAMBA 2050 automated device (SAMBA Technologies, Meylan, Grenoble, France) was performed according to the following protocol.

**Figure 1:**
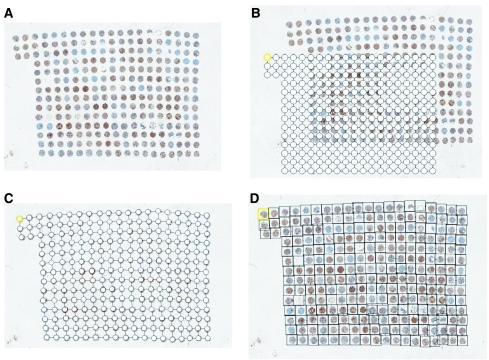
Steps of SAMBA software processing before densitometry on TMA. (**A**) Reference grid, (**B**, **C**) precrop of digitised TMA and surimposed grid (**D**) cropped final image before image analysis (densitometry).

First, an image of the entire slide was built using a low-power magnification (× 2, pixel dimension 3.7 *μ*m). This image was made of a mosaic of images acquired along a rectangular grid with contiguous fields. Second, the area of the slide containing the TMA cores was automatically delineated and scanned at higher magnification (× 10, pixel dimension 7.4 *μ*m). Third, after autofocus, the images were acquired with an overlap greater than the largest mechanical positioning error. Using the image contents, a matching algorithm precisely determined the relative position of each image with respect to its neighbours. Calculated overlap was removed from images to produce a new set of higher magnification (× 10) images, thus covering precisely the cores of interest. A specifically developed tool referred to as TMA crop then allowed superimposition of the TMA grid onto the reduced image and precise alignment of each node of the grid with the core location within the image (Figure 1). The final step was performed automatically using the core image contents to ensure pixel precision of the match. From the images acquired with × 20 magnification, a new set of images was next computed, one for each core. After colour analysis of the core images, SAMBA ‘immuno’ software was applied as previously reported (Charpin *et al*, 1994, 1997a, 1997b, 1998a, 1998b, 1998c, 2004; Dalès *et al*, 2004, 2005) in regular full tissue sections. Several parameters per core were computed: the area of counterstaining, the ratio (as percentage) of the positive area *vs* counterstained areas and a quick score (percentage of positive area × mean optical density). Optical density (OD) was evaluated on a scale of grey levels (arbitrary units) ranging from 0 (100% transmission, OD=0) to 255 (1% transmission OD=2). The computation of each parameter obtained provided numerical values consisting of continuous variables for statistical tests.

### Statistical analysis

Statistical analyses were performed using NCSS 2005 and Statistica Statistical softwares.

The concordance between the immunoexpression data (i) in frozen and fixed tissues, (ii) in full section and TMA sections and (iii) by semiquantitative and automated analysis were examined using Spearman rank correlation. Comparisons of mean parameter values for c-Met, PI3K and E-cadherin were assessed through non-parametric tests. Mean values were computed from measurements in the two cores from each tumour, before correlation between IBC and non-IBC. Contingency table analysis was used to analyse the relationship between protein expression in TMA of the IBC and non-IBC groups (Mann–Whitney, Kolgomorov and *χ*^2^ tests). All *P*-values were two-sided. In the study, mean immunostained surfaces and quick scores were × 10, because absolute values of measurements corresponded to very small numbers.

## RESULTS

### c-Met expression in TMA

**Figure 2:**
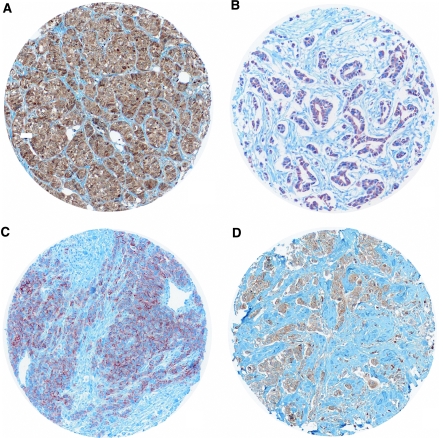
Variation of c-Met cytoplasmic immunostaining stronger in IBC TMA (**A**, **B**) with some focal enhancement beneath the plasma membrane (**C**), than in ductal non-IBC TMA (**D**).

c-Met immunostaining appeared on the tissue sections as dense cytoplasmic staining, irregularly distributed within carcinomatous cells (Figure 2), with focal strong enhancement beneath the plasma membrane.

The c-Met expression was significantly greater in IBC for each parameter assessed by software (Table 1), as shown in particular by a higher mean percentage of immunostained surface (21.3 *vs* 2.7%) (*P*<0.001, Mann–Whitney and Kolgomorov tests) and lower SAMBA quick scores (0.48 *vs* 5.5) *P*<0.001, Mann–Whitney and Kolgomorov tests). The much stronger intensity of staining explained the lower quick scores (arbitrary scale of grey levels: 0 for black to 255 for white).

None of the IBC was c-Met-negative (*n*=0 out of 41), whereas about one-third of the control group was negative (*n*=174 out of 480, *P*<0.001, *χ*^2^).

### PI3K expression in TMA

**Figure 3:**
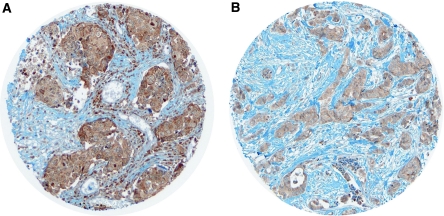
Phosphatidylinositol-3-kinase cytoplasmic immunostaining in (**A**) IBC TMA in (**B**) non-inflammatory ductal carcinoma.

Phosphatidylinositol-3-kinase immunostaining appeared irregularly distributed in the cytoplasm of carcinomatous cells (Figure 3).

The PI3K staining was significantly greater in IBC than in the control group: immunostained surfaces 19.6 and 4.4%, *P*<0.001 and mean quick scores 0.13 *vs* 5.7, *P*=0.0019 (Mann–Whitney). Most IBC were PI3K-positive (*n*=35 out of 41), whereas the positive/negative ratio was lower in the control group (*n*=287 out of 480, *P*<0.01, *χ*^2^).

### E-cadherin expression in TMA

**Figure 4:**
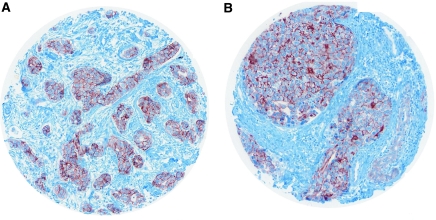
Linear E-cadherin distribution along tumour cell membranes in IBC TMA (**A**, **B**).

E-cadherin was observed along the cytoplasmic membrane (Figure 4).

E-cadherin-positive surfaces, but not the staining intensity (mean optical density), were significantly different between IBC and non-IBC (35.4 and 10.3%, respectively; *P*<0.001, Mann–Whitney) (Table 1). All tumours (ductal carcinomas) were E-cadherin-positive at least focally in both IBC and non-IBC groups (*χ*^2^ test not performed).

## DISCUSSION

Tissue microarrays have been developed to provide cost-effective substrates for high-throughput molecular profiling of tumours and are particularly useful in breast cancer analysis, as shown in several previous reports (Kallioniemi *et al*, 2001; Ginestier *et al*, 2002; Bertucci *et al*, 2004; Charpin *et al*, 2004). However, only one study has recently documented c-Met immunoexpression in TMA from 324 breast carcinomas (Tolgay Ocal *et al*, 2003), showing a worse outcome of node-negative breast carcinomas that overexpressed c-Met.

In the present study using TMA, we confirmed the increased c-Met expression in IBC previously documented by others in non-TMA substrates of aggressive phenotypes of breast cancer (Beviglia *et al*, 1997; Ghoussoub *et al*, 1998). Recent experimental results have shown that the c-Met receptor and c-Met-dependent signalling can be specifically inhibited (Christensen *et al*, 2005), similar to therapies targeting other molecules with, for example, mutant c-Kit in gastrointestinal stromal tumours, trastuzumab in breast cancers overexpressing HER-2, bevacizumab in colorectal carcinoma and genitinib in non-small-cell lung cancer (Birchmeier *et al*, 2003; Christensen *et al*, 2005). However, initial attempts using *in vivo* models of small-molecule therapy, based on inhibition of RTKs by Glivec, Iressa and Tarceva that are used for treatment of multiple human cancers, have revealed a lack of selectivity towards c-Met inhibition (Maulik *et al*, 2002; Ma *et al*, 2003; Pennacchietti *et al*, 2003; Christensen *et al*, 2005; Gasparini *et al*, 2005).

Some approaches specifically to inhibit HGF or c-Met-dependent signalling have been explored (Christensen *et al*, 2003; Laird *et al*, 2003; Matsumoto and Nakamura, 2003; Morton *et al*, 2003; Wang *et al*, 2003; Zheng *et al*, 2003; Schwall *et al*, 2004). Recently, various studies have reported very efficient and selective small molecule inhibitors of c-Met, characterised by the indolin-2-one core structure, such as PH A665752 and Kirin (Christensen *et al*, 2003). Functional assays have indicated that these small molecules inhibit c-Met functions that are dependent on the kinase activity of the receptor, and act upon differential regulation of signalling through inhibition of PI3K or FAK and c-SRC pathways (Christensen *et al*, 2003, 2005; Wang *et al*, 2003). The fact that we found PI3K to be concomitantly overexpressed with c-Met supports the hypothesis that c-Met activation in IBC induces downstream signalling through the PI3K pathway. Collectively, all these data support the notion that c-Met is a potential therapeutic target for either small molecules or biological inhibitors of downstream signal transduction, with biological consequences in tumour cells that would be of value in the management of patients with IBC.

Loss of epithelial cell adhesion molecules of carcinomatous cells is also partly responsible for tumour spreading. E-cadherin is a transmembrane protein that mediates cell–cell adhesion in epithelial tissues. It connects epithelial cells through its association with catenins, which anchors E-cadherin to cells in the cytoskeleton, and thus aids in maintaining cell–cell adhesion (Birchmeier *et al*, 2003; Christensen *et al*, 2005; Gasparini *et al*, 2005). Hepatocyte growth factor/scatter factor stimulation reduces *β*-catenin binding to the intracellular portion of E-cadherin, resulting in loss of cell–cell adhesion (Birchmeier *et al*, 2003; Christensen *et al*, 2005; Gasparini *et al*, 2005). Increased HGF/c-Met stimulation may therefore play a role in altering cell adhesion in human cancers overexpressing c-Met. In breast carcinomas, mutation of the gene encoding E-cadherin is responsible for low expression of the protein in tumours associated with a poor outcome and frequent remote metastases Charpin *et al*. (1998c). Lobular carcinomas are now well recognised as tumours having a mutation of the gene encoding E-cadherin, a lack of E-cadherin immunohistochemical expression, and a particular phenotype and microscopic pattern with a typical permeation throughout the breast tissue by isolated cells and small nests of tumour cells. In contrast, IBC have surprisingly been reported to overexpress E-cadherin see review in (Charafe-Jauffret *et al*, 2004). Similarly, in our study, IBC exhibited significantly increased E-cadherin expression. Overall, these data suggest that in IBC, overexpression of c-Met may act upon cell adhesion and spreading in spite of preserved or even overexpressed E-cadherin. The overexpressed E-cadherin is either inefficient or more likely retains strong homotypic tumour cell–tumour cell adhesion capacity, producing compact spheroids, while tumour cell–endothelial cell aversion develops, favouring dissemination of tumour emboli. This aversion has been reported to result from non-functional cytoplasmic glycosylates (MUC1) with subsequent lack of tumour-cell binding to E-selectin at the endothelial surface (Bertucci *et al*, 2004; Charafe-Jauffret *et al*, 2004).

Developments in research and routine practice in pathology have followed progress in molecular biology, permitting the development of TMA for validation of immunocytochemical assays in a large number of tumours (Kallioniemi *et al*, 2001; Ginestier *et al*, 2002). Nevertheless, construction of TMA requires (a) some technical experience to evaluate available tumour markers for widespread use, (b) the availability of large tumour libraries to allow documentation of marker expression in tissues and (c) the ability of pathologists to select archival tissues and to collect patient follow-up data. Although semiquantitative evaluation of TMA is very difficult and time-consuming for pathologists and may be a source of non-reproducible interpretation using arbitrary methods for scoring of immunostaining and lack of quality control, limited data are available in the literature concerning the automated quantification of immunoprecipitates on TMA (Camp *et al*, 2002; Pages *et al*, 2006). However, appropriate software dedicated to immunocytochemical tissue labelling, along with the latest image analysis devices, can considerably facilitate the rapid, high-throughput measurement of immunostaining in hundreds of immunostained cores within TMA tissue sections, with completely automated digitisation of microscopic slides (up to 50 slides per batch) in only a few hours. Such combinations of hardware and software allow quantified assessment of immunoprecipitates, providing continuous values for the usual parameters of measurement suitable for statistical computation, and are also particularly helpful for distinguishing minor differences in staining surfaces and intensities that are nearly impossible to detect by a pathologist on microscopic examination (Charpin *et al*, 1994, 1997a, 1997b, 1998a, 1998b, 1998c, 2004).

On the basis of our experience with image analysis, we have developed TMA suitable for automated quantitative analysis and applied dedicated software to densitometry in TMA in order to compare expression of c-Met and downstream transduction signals like PI3K immunohistochemically in IBC and non-IBC. Our results confirm that c-Met and PI3K are significantly overexpressed in IBC, suggesting that in this particular clinical setting, c-Met expression and downstream signal transducers may be regarded as potential targets for specific therapies. Also, overexpression of c-Met as well as E-cadherin appears to belong to the proteomic signature of IBC.

## Figures and Tables

**Table 1:** Immunohistochemical expression of c-Met, PI3K, E-cadherin in IBC (inflammatory breast carcinomas) and in noninflammatory control ductal breast carcinoma series, measured by automated densitometry through image analysis, SAMBA 2050 (mean values × 10/^*^Mann–Whitney test; ^**^χ^2^ tests).

	**Immunostained surface (%)**	**SAMBA quick score**	**Pos/neg cases**	
	**Mean**	**Mean**	**Positive**	**Negative**
*c-Met*				
Inflammatory breast cancers (*n*=41)	21.3% (s.d.=2.6)	0.48 (s.d.=0.18)	41/41	0/41
	^*^P<0.001	^*^P<0.001	P^**^<0.001	
Control series of non-inflammatory breast carcinomas (*n*=480)	2.7% (s.d.=0.81)	5.5 (s.d.=0.53)	306/480	174/480
				
*PI3K*				
Inflammatory breast cancers (*n*=41)	19.6% (s.d.=2.4)	0.13 (s.d.=0.002)	35/41	06/41
	^*^P=0.0019	^*^P<0.001	P^**^<0.01	
Control series of non-inflammatory breast carcinomas (*n*=480)	4.4% (s.d.=38)	5.7 (s.d.=0.002)	287/480	193/480
				
*E-cadherin*				
Inflammatory breast cancers (*n*=41)	35.4% (s.d.=5.2)	5.2 (s.d.=2.1)	41/4	0/41
	^*^P<0.001	^*^P=NS		
Control series of non-inflammatory breast carcinomas (*n*=480)	10.3% (s.d.=1.04)	6.7 (s.d.=2.09)	480/480	0/480

Abbreviations: PI3K=phosphatidylinositol-3-kinase; s.d.=standard deviation.
